# Use of Subtherapeutic Tylvalosin Against *Mycoplasma hyopneumoniae*: Implications For Respiratory Microbiome Dysbiosis and Swine Lung Health

**DOI:** 10.1155/tbed/8903237

**Published:** 2025-08-18

**Authors:** Leonardo Teófilo Toledo, Richard Costa Polveiro, Caio Augustus Diamantino, Gustavo Manoel Rigueira Simão, Carlos Eduardo Real Pereira, Eduardo de Freitas Costa, Maria Aparecida Scatamburlo Moreira, Fernanda Simone Marks

**Affiliations:** ^1^Department of Veterinary Medicine, Federal University of Viçosa, Viçosa 36570-900, Minas Gerais, Brazil; ^2^Faculty of Veterinary Medicine and Animal Science, Federal University of Uberlândia, Uberlândia 38410-337, Minas Gerais, Brazil; ^3^Agroceres PIC Company, Rio Claro 13502-741, São Paulo, Brazil; ^4^Department of Epidemiology, Bioinformatics and Animal models, Wageningen Bioveterinary Research, Lelystad, Flevoland, Netherlands

**Keywords:** antibiotics, enzootic pneumonia, experimental challenge, microbiota, respiratory disease, UFV01

## Abstract

Enzootic pneumonia (EP) caused by *Mycoplasma hyopneumoniae* (*M. hyopneumoniae*) has a significant impact on swine production. Subtherapeutic exposures of tylvalosin in swine, often due to inconsistent dosing in feed or water, promote antimicrobial resistance. This study investigated the efficacy of 1.0625 mg/kg/day of tylvalosin administered for 7 days via feed to pigs experimentally infected with the UFV01 strain of *M. hyopneumoniae* and its impact on the respiratory microbiome. Thirty landrace x large white female piglets were divided into three groups: G1 (negative control, *n* = 2), G2 (infected, *n* = 14) and G3 (infected and treated, *n* = 14). Clinical signs, seroconversion, macroscopic and microscopic lung lesions and bacterial load were assessed. The respiratory microbiota of swine was analysed through 16S rRNA gene sequencing, followed by bioinformatics analyses. While G1 piglets remained healthy, G2 and G3 piglets developed lung lesions consistent with EP, although no significant difference was observed between these groups. Seroconversion was higher in G2 (90.9%) than in G3 (45.5%) at 35 days post-infection, suggesting modulation of the humoral immune response by tylvalosin. Microbiota analyses revealed a significant shift in post-infection composition, with infected pigs exhibiting reduced alpha diversity and distinct beta diversity compared to healthy pigs. *M. hyopneumoniae* dominated the respiratory microbiome of infected animals, drastically reducing the abundance of other taxa, notably *Stenotrophomonas maltophilia*. While tylvalosin treatment partially restored alpha diversity and shifted the microbiota composition towards the control group, it failed to eliminate *M. hyopneumoniae*. *Variivorax*, *Ralstonia* and *Pseudomonas* were identified as potential biomarkers for respiratory health and treatment response. These findings emphasise the complex relationship between *M. hyopneumoniae* infection, suboptimal tylvalosin dosage and resulting respiratory microbiome dysbiosis. Identifying and correcting the inappropriate use of antimicrobial dosages in clinical and preventive treatments, as well as promoting research focused on optimising dosage strategies and management practices, is essential for swine production and for reducing antimicrobial resistance. Moreover, maintaining a balanced microbiota may be a key factor in achieving healthier swine production, both in terms of animal welfare and food safety for consumers.

## 1. Introduction

*Mycoplasma hyopneumoniae* (*M. hyopneumoniae*) is the primary causative agent of enzootic pneumonia (EP), a chronic respiratory disease in pigs characterised by a dry cough, reduced weight gain, high morbidity and low mortality [1, 2] and one of the main pathogens involved in the porcine respiratory disease complex (PRDC) [1]. Bacterial pathogens, such as *Pasteurella multocida*, *Streptococcus suis*, *Actinobacillus pleuropneumoniae* and *Glaesserella parasuis*, are frequently identified in co-infections with *M. hyopneumoniae* [3–10]. However, the presence of these pathogens does not invalidate the central role of *M. hyopneumoniae* in the pathogenesis of swine respiratory disease. On the contrary, *M. hyopneumoniae* is widely recognised as a primary pathogen that invades the respiratory epithelium and facilitates secondary infections [1–7]. Several studies have shown that *M. hyopneumoniae* infection significantly exacerbates pulmonary lesions in coinfections with these secondary agents [8]. Microbiota analyses have also confirmed the presence of *P. multocida* in clinically healthy pigs free of *M. hyopneumoniae* but with high bacterial abundance [10].

Cases of EP lead to significant economic losses for the global swine industry [2, 3]. Despite increased investment in biosecurity measures, antibiotics are still frequently employed in the treatment of EP in swine. However, this practice should be carefully evaluated on a case-by-case basis, considering alternatives and prioritising responsible use with appropriate dosages and durations to minimise the development of antimicrobial resistance [11, 12].

Antibiotic usage in animals is frequent not only for treatment, but also to control the spread of infections (metaphylaxis) and prevent infections (prophylaxis), especially during periods of stress and increased vulnerability. Antibiotics are also used to improve feeding efficiency and promote growth [13]. Tylvalosin is a third-generation macrolide antibiotic with broad-spectrum antibacterial activity. It is effective against mycoplasmas and Gram-positive bacteria and inhibits some Gram-negative bacteria [14]. It has been proven to be effective for treatment of EP, significantly reducing clinical signs and bacterial load in the lungs of infected animals [15]. Its administration is also associated with improvements in weight gain and feed assimilation efficiency, contributing to overall animal health and the productivity of swine farms [7].

Tylvalosin is widely used in the treatment and metaphylaxis of bacterial infections in livestock. Derived from tylosin, this antibiotic is known for its efficacy against a range of respiratory and enteric pathogens, particularly in pigs and poultry [16, 17]. Interestingly, macrolides have been reported to have immunomodulatory effects [18], and some studies have demonstrated tylvalosin efficacy against viruses, such as porcine reproductive and respiratory syndrome virus [17, 19–21] and bacteria such as *M. hyopneumoniae* [14, 21], *P. multocida* [14] and *Lawsonia intracellularis* [22]. Its anti-inflammatory properties have been observed in different infection models, decreasing the concentrations of interleukin (IL)-8, IL-6, IL-1β and tumour necrosis factor (TNF)-α in vitro or in vivo [18, 20].

Tylvalosin is usually administered via drinking water or feed, facilitating its large-scale application in intensive production systems [14, 18]. Dosage and treatment duration vary depending on the animal species and the nature of the infection. Subdosing of antibiotics can occur in treatment, metaphylaxis and prophylaxis due to dosage errors, improper administration or flaws in management practices. The difficulty in ensuring accurate antimicrobial dose administration can spoil the application of uniform and predictable doses. It is influenced by several often-overlapping factors, such as mislabelling, veterinary supervision, feed characteristics, livestock worker behaviour, animal behaviour and the pharmacokinetics of the drug [23].

Inadequate or inconsistent drug administration can lead to treatment failures [24] or the emergence of antimicrobial-resistant strains in livestock [25–27]. Furthermore, subdosing may fail to achieve the therapeutic concentration necessary for complete pathogen elimination, promoting the selection of resistant bacteria and compromising the long-term efficacy of antimicrobial treatments. Additionally, the uneven distribution of the drug in collective delivery systems, commonly observed in these practices, exacerbates the risk of inadequate exposure, enabling bacteria to develop and disseminate resistance mechanisms, such as target-site modifications and increased efflux [28, 29]. Subtherapeutic doses are illegal in many countries [30, 31]. This ban is based on studies, similar to what we describe here, that show the harmful effects of the practice. Accumulating evidence indicates that it is crucial to strengthen regulations in countries where the practice still persists [32].

Recent studies on swine have employed tylvalosin administered via water, targeting a dose of 5 mg/kg/day in the final third of gestation in sows and 20 mg/kg/day for 10 days in piglets aged 3–4 weeks [21]. Another study used 10 mg/kg/day for five consecutive days, also via water [14]. Aivlosin is registered with the European Medicines Agency [16] (EMA), which recommends a tylvalosin dose of 2.125 mg/kg/day for 7 days for *M. hyopneumoniae*. Although underdosage is a common occurrence, very little is known about the effects of subtherapeutic dosing.

Our study aimed to evaluate the impact of tylvalosin subdosing (1.0625 mg/kg/day via feed for 7 days) in controlled experimental infections of pigs with *M. hyopneumoniae*. Parameters of clinical signs, seroconversion, lesions, bacterial load and the effect of subdosing on treatment and the respiratory microbiota of infected animals were analysed. This research is crucial for understanding the efficacy of suboptimal antibiotic dosages in combatting infections and their impact on modulating the resident microbiota, with direct implications for animal health. Understanding how *M. hyopneumoniae* and tylvalosin subdosing affects the lower respiratory tract microbiota is essential for unravelling the microbial interactions that can lead to disease development. The use of suboptimal doses of antibiotics constitutes antimicrobial misuse [30], which veterinary professionals should actively avoid. Subdosing likely contributes to the development and spread of antimicrobial resistance [31].

## 2. Materials and Methods

### 2.1. Origin and Cultivation of UFV01 Strain

The UFV01 strain (GenBank accession: PRJNA542605) was isolated from a property located in Vale do Piranga, Minas Gerais, Brazil and was later characterised [33]. This isolate is characterised as highly pathogenic, thus making it suitable for animal challenge in the current study.

Friis medium was prepared largely as described [34]. To make 500 mL of Friis medium, 1.5 g of brain heart infusion (BHI, Kasvi) and 1.6 g of pleuropneumonia-like organisms (PPLO) broth (Himedia) were dissolved in 365 mL of water and sterilised by autoclaving. To this were added 18 mL of yeast extract (prepared from dried baker's yeast), 12.5 mL of sterile solution A (160 g/L NaCl, 4, 8 g/L KCl, 2 g/L MgSO_4_·7H_2_O, 2 g/L MgCl_2_·6H_2_O, 3.7 g/L CaCl_2_·2H_2_O), 12.5 mL of sterile solution B (3.0 g/L Na_2_HPO_4_·12H_2_O, 1.2 g/L KH_2_PO_4_), 50 mL of pig serum (Invitrogen) heat-treated at 56°C for 20 min, 50 mL of heat-treated horse serum (Invitrogen) and 1 mL of phenol red solution (0.6% in 0.1 M NaOH). The solution was adjusted to pH 7.4 with 1.0 M NaOH. Antibiotics were not incorporated as recommended [35]. Solid culture Friis medium (2.8x) was prepared by the addition of 88 mL of water to 4.3 g BHI, 4.6 g of PPLO broth sterilised by autoclaving, 51.4 mL of yeast extract, 35.7 mL of sterile solution A, 35.7 mL of sterile solution B, 143 mL of heat-treated horse serum, 143 mL of heat-treated pig serum, 2.8 mL 0.6% of phenol red solution and adjusted to pH 7.4. Friis agar was prepared by mixing concentrated Friis medium (2.8x) at a ratio of 35:65 with 1.8% agar (Oxoid) [34].

The strain *M. hyopneumoniae* (UFV01) were reactivated in a liquid medium at a ratio of 1:9 (frozen inoculum to medium). The test tubes were incubated at 37°C and observed daily until a colour change occurred. Bacterial growth was evidenced by the alteration of the medium's colour. Three passages were performed to reactivate the isolate, and after the fourth passage, the inoculum was aliquoted into individual volumes of 7 mL and frozen at −80°C until the time of inoculation. The isolates were quantified using the colour changing units (CCUs) technique [35]. The recommended CCU was 10^7^/mL [33]. A sterility test was performed on the cold medium and the UFV01 strain inoculum by inoculating both blood agar and BHI agar plates. Both were negative for growth.

### 2.2. Experimental Design and Sample Collection

The study was conducted at the experimental unit of the Department of Veterinary at the Federal University of Viçosa. Thirty 21-day-old female pigs (landrace x large white) were obtained from Agroceres PIC (Brazil). The animals come from a genetic multiplication farm of the company, where their negativity for *M. hyopneumoniae* has been proven for 24 years. The animals were acclimatised for 10 days prior to inoculation (day-10 to day-0). During acclimatisation, laryngeal swabs were collected as described below. All animals tested negative for *M. hyopneumoniae* by quantitative polymerase chain reaction (qPCR) [33, 36] and for *M. hyopneumoniae* immunoglobin G (IgG) antibodies by using a commercial enzyme-linked immunosorbent assay (ELISA) kit (IDEXX, USA). Complete blood counts and biochemistry panels were performed to assess the animals' initial health status.

**Figure 1:**
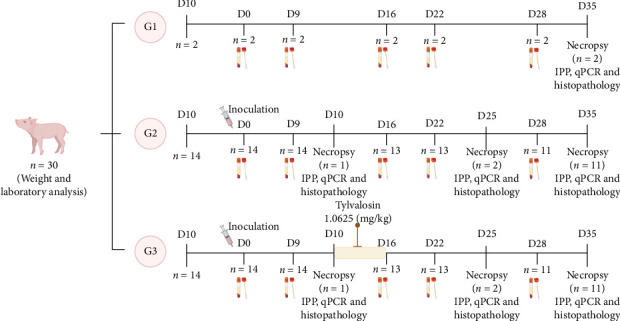
Experimental design chart. G1, G2 and G3 are the experimental groups. D shows the day of indicated experimental processes, such as sampling (blood draw and laryngeal swabbing) and inoculation with *M. hyopneumoniae*.

For the experimental design, we balanced the animals' weight for group formation to ensure homogeneity among the experimental groups. The pigs were divided into three groups:

i. Negative control (G1, *n* = 2): animals inoculated with 7 mL of Friis medium and placed in an isolated barn.
ii. Positive control (G2, *n* = 14): animals received an intratracheal inoculation [33] of 7 mL of UFV01 strain at 10^7^ CCU/mL.
iii. Test group (G3, *n* = 14): animals received an intratracheal inoculation of 7 mL of UFV01 strain at 10^7^ CCU/mL and treated with 1.0625 mg/kg/day of tylvalosin in feed daily for 7 days (days 10–16 in Figure 1).

The EMA recommends a therapeutic dose of tylvalosin of 2.125 mg/kg of active pharmaceutical product for 7 days for *M. hyopneumoniae* [16]. To reproduce the effect of subdosing, our study used a commercial product with a tylvalosin concentration of 50% at a dose of 2.125 mg/kg/day, resulting in a subtherapeutic dosing of tylvalosin at 1.0625 mg/kg/day. From 7 to 9 days post-inoculation (dpi), the daily feed intake of G3 piglets was measured to estimate the average consumption. On day 10 dpi, all animals in G3 were placed in individual pens to allow precise control of medicated feed intake. The daily tylvalosin dose for each pig was individually weighed in the laboratory using a precision balance and pre-mixed with 100 g of feed to ensure homogenous distribution, given the small volume of active compound. At feeding time, this medicated portion corresponding to 25% of the estimated daily feed intake was first offered. Only after complete consumption of this portion was the remaining 75% of the feed, without antibiotic, provided. Animal weights used to calculate the dosage were measured at 9 dpi (1 day before the start of treatment) and again at 12 dpi (to adjust dosing during the treatment period). After the 7-day treatment period, the animals were returned to group housing. Animals in G1 and G2 received feed without tylvalosin. All groups received feed formulated to meet the requirements of the growth phase according to the Brazilian Tables for Swine and Poultry [37].

On the day of inoculation (D0) and at 9, 16, 22, 28 and 35 dpi), serum and laryngeal swab samples were collected from all animals to assess the seroconversion curve and *M. hyopneumoniae* excretion (Figure 1). Laryngeal swabs were collected by inserting a sterile swab (BBL CultureSwab, Sparks, MD, USA) into the oral cavity and advancing it to the larynx with the aid of a laryngoscope. Once the larynx was visualised and the epiglottis was in a lowered position, the internal walls of the laryngeal cartilages were gently swabbed. All swabs were initially kept at 4°C after collection, transported to the laboratory, and stored at −80°C until processing and testing.

At 10 dpi, two animals were randomly selected, one from each of G2 and G3, and euthanised to confirm pre-treatment infection. Macroscopic and microscopic lesions were assessed and the *M. hyopneumoniae* bacterial load was determined by qPCR in bronchoalveolar lavage fluid (BALF) and lung tissue. Re-isolation of the UFV01 strain was performed [34] and identification confirmed by qPCR [36]. Subsequently, at 25 dpi (9 days after the end of treatment), two animals from each of G2 and G3 were randomly selected for euthanasia and sample collection to evaluate the course of post-treatment infection. At the end of the experiment (35 dpi), all remaining animals were euthanised, and samples lung tissue and BALF were collected (Figure 1).

During necropsy BALF samples were collected using a sterilised glass pipette. Briefly, 15 mL of sterile phosphate buffered saline (PBS) solution (pH 7.4) (Sigma-Aldrich, USA) was instilled into the trachea near the bifurcation into the main bronchi. The lungs were then gently massaged to ensure adequate distribution of the fluid throughout the airways. Subsequently, the lavage fluid was aspirated using an automatic pipettor and transferred into 1.5 mL cryotubes free of DNases and RNases (Corning, USA). Samples were immediately stored at −80°C until further analysis.

### 2.3. Clinical Evaluation and Cough Frequency

Monitoring of piglets in all groups was conducted twice daily for 30 min. The incidences of dry cough and sneezing episodes during this period were systematically quantified and documented by a single blinded observer [38]. Clinical evaluations were performed on all animals 10 days before inoculation, on D0, and at 3, 5 and 9 dpi (before antibiotic treatment commencement), 16 dpi (end of treatment) and 22, 28 and 35 dpi (end of study). Clinical examinations assessed respiratory rate, heart rate and rectal temperature [39, 40].

### 2.4. ELISA for Detection of Anti-*M. hyopneumoniae* IgG

Blood samples were collected immediately before *M. hyopneumoniae* inoculation and at 9, 16, 22, 28 and 35 dpi. The blood samples were centrifuged at 8000 × *g* for 15 min and 1 mL aliquots of serum were stored at −20°C until testing.

Detection of *M. hyopneumoniae*-specific IgG antibodies in the serum was performed using a commercial indirect ELISA kit with 99.6% specificity and 89.4% sensitivity (IDEXX *M. hyopneumoniae* Ab test, USA) [33, 41]. The assay was conducted according to the manufacturer's instructions, and absorbance readings were measured at 650 nm with a SpectraMax M5 Multi-Mode Microplate Reader (Molecular Devices, USA). As the animals were experimentally infected and maintained in isolated facilities, sample-to-positive (S/P) ratios above 0.3 were considered positive [33, 41]. The S/P ratio represents the relative optical density of the sample compared to controls, providing a quantitative measure of antibody levels or antigen presence in the assay.

### 2.5. Detection of *M. hyopneumoniae* DNA by Real-Time qPCR

DNA was extracted from the laryngeal swab, lung tissue and BALF samples by using a Wizard Genomic DNA Purification Kit (PROMEGA, USA) following the manufacturer's recommendations. As endogenous controls for the DNA extractions from the samples, a primer that amplifies a 107 bp region of the 18S ribosomal gene (GenBank: AY265350.1) [42] was used.

For the detection of the *M. hyopneumoniae* genome from adhesin p102 in the samples, the set of primers and probe comprising forward primer 5-′ TAAGGGTCAAAGTCAAAGTC-3′, reverse primer 5′- AAATTAAAAGCTGTTCAAATGC-3′ and hydrolysis probe 5′-FAM-AACCAGTTTCCACTTCATCGCC-BHQ2-3′ [36]. A fragment of *M. hyopneumoniae* DNA was amplified by conventional PCR and cloned using the CloneJET PCR Cloning Kit (Thermo Fisher) for the construction of the standard curve. All samples were tested in duplicate, and those showing variation greater than 0.5 Ct were tested again in triplicate. The reaction consisted of 10 μL of 2x iTaqTM Universal Probes Master Mix (Bio-Rad, city, CA, USA), 1 μL of each primer at 10 pmol/μL (Invitrogen, USA), 0.6 μL of the hydrolysis probe at 10 pmol/μL (IDT, Iowa City, IA, USA), 5.4 μL of ultrapure water and 2 μL of DNA per sample, totalling 20 μL of reaction. The qPCR was performed on a CFX-96 real-time thermocycler (Bio-Rad, city, CA, USA) with an initial denaturation cycle at 95°C for 3 min, followed by 39 cycles of 95°C for 15 s per cycle and annealing/extension at 55.7°C for 1 min [41].

### 2.6. Macroscopic and Microscopic Lesions

After the euthanasia of the animals, lung lesions compatible with swine EP were scored [43]. Each of the lung lobes was assessed macroscopically and the percentage of surface area affected by lesions was estimated. Subsequently, this percentage was multiplied by the weight of each lung lobe to estimate the proportion of the lung affected by lesions, ranging from 0% (no lesions) to 100% (entire lung affected). The evaluated characteristics included red and firm consolidation observed in the apical, cardiac, accessory and diaphragmatic lobes.

Histological slides were prepared for semiquantitative classification of microscopic lesions [44]. The sections were systematically examined by evaluating the following structures in each section: bronchi, bronchioles, bronchus-associated lymphoid tissue (BALT), alveolar ducts and alveoli, including alveolar septa, peribronchial, peribronchiolar and interlobular connective tissues and pleura. BALT hyperplasia was graded as: (−) absent; (1) mild, diffuse infiltration of lymphocytes in peribronchial, peribronchiolar and perivascular tissues, including the lamina propria of the airways; (2) moderate increase in diffuse lymphocyte infiltration and/or presence of some lymphoid nodules; or (3) intense, with marked the number of lymphoid nodules. Bronchopneumonia and pleuritis lesions were graded in the same way, according to the intensity of the lesions [44]. The pathologists were blinded to sample origin for assessing clinical symptoms and lesions.

### 2.7. 16S rRNA Gene Amplification and Sequencing

A total of 32 BALF samples were used for respiratory microbiota analysis. Of these, 30 samples were collected from pigs in the experimental groups G1, G2 and G3 at the time of euthanasia, at the end of the trial. In addition, two supplementary samples were included from *M. hyopneumoniae*-negative control animals of the same age and genetic background, which were maintained under identical experimental conditions as part of a separate study previously conducted by our research group. The inclusion of these two samples aimed to enhance the representativeness of the control group in the respiratory microbiota analysis, allowing for a more accurate characterisation of the microbial profile of pigs not infected with *M. hyopneumoniae*, and serving as a reference for comparisons with the experimentally challenged groups.

The total DNA of the BALF samples was extracted using the PureLink Genomic DNA Mini Kit (Thermo Fisher Scientific, USA) and stored at −80°C. The concentration and purity of the DNA were quantified by two different methods, the first by spectroscopy (optical density) in a Nanodrop Thermo Fisher Scientific spectrophotometer (Waltham, MA, USA) and the second through the Qubit dsDNA BR kit, with a Qubit 2.0 Fluorometer (Life Technologies, Carlsbad, CA, USA).

Samples of extracted DNA were lyophilised in an Edwards vacuum lyophiliser and sent in an isothermal box, duly identified for sequencing, to the Beijing Genomics Institute (BGI) in China. The libraries were prepared using BGI protocol. Briefly, two rounds of PCR were conducted using the MGI ATOPlex 16S panel (MGI, Shenzhen, China). The DNB libraries were sequenced on an MGI DNBSEQ-G400 (MGI, Shenzhen, China) in paired-end sequencing (2 × 300 bp/PE300). The V3/V4 hypervariable regions of the 16S rRNA gene were amplified from genomic DNA by PCR using primers 341F (CCTACGGGRSGCAGCAGCAG) [45] and 806R (GGACTACHGGGGTGGCTAAT) [46].

### 2.8. Bioinformatics Data Processing and Analysis

The analyses followed pipelines executed in the R programming language. Initially, the adapter sequences were removed using the Cutadapt v4.9 software [47]. Afterward, the amplicon sequence variants (ASVs) were inferred using the 'DADA2' (version 1.20.0) package in R [48, 49] (https://benjjneb.github.io/dada2/tutorial.html). The analysis parameters were truncLen = 260 and maxEE = 3,5. The 'chimaera' sequences identified for each sample were removed by the consensus method. The taxonomic assignment was performed at the genus and species level according to the pipeline described with the DADA2 workflow (https://benjjneb.github.io/dada2/). The databases used were Silva v.138.1 [50]. The search and classification were performed using standard parameters by implementing the Ribosomal Database Project Classifier (RDP), a Bayesian classifier [51, 52].

The Phyloseq package [53] was used to visualise and filter potential contaminants from the ASV dataset. Potential contaminants among the ASV resulting from sample processing were also eliminated. The final dataset of non-bacterial ASV and ASV without phylum assignment, as well as those with phylogenetic attributes related to the kingdom Archaea and the families chloroplast and mitochondria, were removed. We applied a prevalence and abundance threshold for bacterial ASV of 1% ASV in at least one sample. The rarefaction curves and distribution of reads by phylum were calculated using the microbiome utilities package [54]. The microbiota composition was analysed using bar graphs generated by the Phyloseq package (version 3.19) [55] and ggplot2 (version 3.5.1) [56].

The alpha diversity (within-sample evenness and/or richness) was analysed in Phyloseq [53], using six indices that were used to measure alpha diversity, including community richness (observed species, Chao1 and analysing, composing and evaluating (ACE) [56], and community diversity (Shannon and Simpson indices) [57, 58]. The values of the indices for the different groups-infected (G2 and G3 at 10 dpi), treated_25 (G3 at 25 dpi), infected_25 (G2 at dpi 25), treated_35 (G3 at 35 dpi), infected_35 (G2 at 35 dpi), and control (G1)-were compared using ggplot2, Vegan (version 2.6-6.1) and microbiome packages in Phyloseq [53, 55, 59, 60].

Beta diversity (between-sample diversity or community structure) was analysed with Phyloseq [53] and microbial package version 0.2 in R (https://github.com/guokai8/microbial). The final principal coordinate analysis (PCoA) plot was visualised, illustrating the separation of microbial communities based on disease status and the number of dpi. Ellipses around the points indicated the variation within each group, allowing for visual comparison of the beta diversity among the groups infected, treated_25, infected_25, treated_35, infected_35 and control.

Differential relative abundance was analysed using the ALDEx2 package [61], applying a *t*-test on centred log-ratio-transformed genus-level data, while accounting for sample type variance. The Benjamini-Hochberg procedure corrected *p*-values under 0.05. The ALDEx2 package also provided the expected effective size for the paired sample types. Comparisons were made between the infected, treated and control groups (infected vs. control, treated_25 vs. control, treated_35 vs. control and infected_35 vs. control). Data visualisations were created using the microViz [62] and ggplot2 R packages and the Venn diagram was constructed with VennDiagram (version 1.7.4).

### 2.9. Data Availability

The DNA sequences generated and analysed during the current study are available in the National Centre for Biotechnology Information's Sequence Read Archive repository under BioProject PRJNA1146902.

### 2.10. Differential Diagnosis

Individual lung BALF were collected from the animals and subjected to total RNA extraction using the commercial RNA extraction kit from Promega USA-ReliaPrep RNA Miniprep Systems for the diagnosis of the influenza A virus used conventional reverse transcription (RT)-PCR methodology. The primers used in the RT-PCR reactions as a reference were described by Fouchier et al. [63]. For the detection of the bacterial agents (*G. parasuis*, *P. multocida*, *Mycoplasma hyorhinis*, *S. suis* and *A. pleuropneumoniae*), the qPCR technique was used with DNA samples obtained from the BALF [36, 64–68].

### 2.11. Statistical Analysis

Two different generalised linear mixed models [69] employing an identity link function (i.e., normal residual distribution) were set to explore the effect of treatments in G2 and G3 on the outcomes of S/P ratio (from ELISA titres) and copy numbers/μL (from qPCR) from samples obtained via nasal swabs on D0 and 9, 16, 22, 28 and 35 dpi. The copy numbers/μL were log-transformed (after adding 1) to better fit the linear model. The linear predictor of the model included groups (G2 vs. G3) as fixed effects. For the ELISA titres, animals were treated as random effects (i.e., repeated measures), while in the copy numbers model, the random effect accounted for replication nested within each animal. Random effects were assumed to be independently and normally distributed around zero with respective variance components. Inference was based on restricted maximum likelihood, as implemented in R [70] using the lmer function from the lme4 package [71]. Pairwise comparisons between groups were performed using a Tukey-type HSD test, controlling experiment-wise error, as implemented in the R package emmeans [72].

Two additional generalised linear mixed models were used to explore the effect treatment in G2 and G3 on the pleuropneumonia index (PPI%) and copy numbers per μL (from qPCR) extracted from bronchotracheal washes and lung tissue at 35 dpi only. These models were specified in a similar manner to those mentioned above. For PPI%, there were no repeated measures over time, and no random effects were included. In the copy numbers model, animals were treated as random effects to account for the effect of two replicates per animal. The models were implemented as described above.

The histologic lesions of bronchopneumonia, BALT hyperplasia and pleuritis were assessed as ordinal scores (0, 1, 2 and 3). At 35 dpi, the model was evaluated using a cumulative link model [73] with a logit link function (i.e., an ordered logit model). The linear predictor of the model included G2 vs. G3. Approximate maximum likelihood inference was based on Laplacian integration, as implemented in R [70] using the clm function from the ordinal package [74, 75].

## 3. Results

### 3.1. Animal Assessment

Over the observation period, several significant differences (*p* < 0.05) were observed within each group. Rectal temperatures remained within the reference range of 40°C as established by Liedel et al. [76] (Supporting Information 1: Table S1). Although some fluctuations in body temperature were observed, they remained within normal physiological limits and did not indicate a febrile response. These variations in the body temperature may be attributed to handling stress, environmental conditions or normal circadian rhythms, rather than to a febrile infectious condition. Similar temperature fluctuations over time have also been reported by Storino et al. [77] and Sonalio et al. [78].

With regard to heart and respiratory rates, there is a lack of well-established reference standards for pigs within this age range and under the experimental conditions evaluated in this study. This limitation led us to perform comparisons exclusively between the experimental groups, in which only minor differences were observed. Moreover, factors such as animal handling, ambient temperature and the circadian cycle may significantly influence these physiological parameters at the time of measurement, which could explain the slight variations observed throughout the experiment.

### 3.2. Laboratory Evaluation (Erythrogram/Biochemical)

The laboratory analysis results are presented in Supporting Information 2: Table S2. It is important to note that values can vary significantly depending on the analysis conditions and the laboratory. Furthermore, several factors can influence blood composition, including time of collection, genetic factors, age, sex, nutrition and environmental and physiological conditions including stress and excitement [79].

A complete blood count was performed prior to the start of the study to certify the health status of the animals. No significant differences (*p* > 0.05) were observed between the groups for haematological variables or for the biochemical parameters aspartate aminotransferase, alkaline phosphatase, total bilirubin, indirect bilirubin, gamma-glutamyl transferase, albumin, globulin, total protein, creatinine and urea. However, significant differences (*p* < 0.05) were found in alanine aminotransferase and direct bilirubin levels, both of which were higher in G1 than in G2 and G3 (Supporting Information 2: Table S2). Nevertheless, all values were within the normal reference ranges established by the Clinical Pathology Laboratory of our university, based on international standards [80, 81]. All animals were in good health and considered fit to participate in the study.

### 3.3. ELISA and qPCR

**Figure 2:**
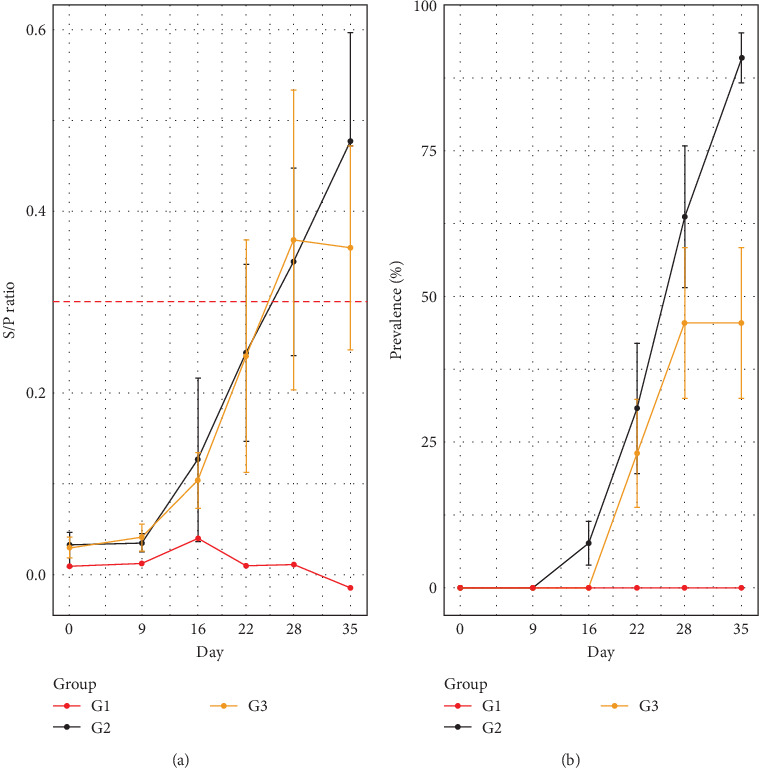
(A) Seroconversion dynamics for *Mycoplasma hyopneumoniae* IgG antibodies in pigs experimentally infected with the UFV01 strain over time (days post-inoculation [dpi]). Sample-to-positive (S/P) ratio: the S/P ratio represents the relative optical density of the sample compared to controls, providing a quantitative measure of antibody levels or antigen presence in the assay. (B) Seroconversion for *M. hyopneumoniae* IgG antibodies in pigs 35 days after experimental inoculation with the UFV01 strain. The lines represent the percentage of seroconverted animals in each treatment group.

Statistical analysis of the linear model revealed a significant positive effect at 22, 28 and 35 at on the S/P ratio, indicating a consistent increase in S/P values on these days for pigs in G2 and G3. Conversely, there was no statistical significance at 9 and 16 dpi, suggesting no significant change in the S/P ratio during these periods. No significant differences were observed between G2 and G3, nor for their interactions with the time points, indicating that the S/P ratio did not differ between groups over time (Figure 2A).

Figure 2 reveals distinct seroconversion dynamics between G2 (infected) and G3 (infected/treatment). G1 remained negative for IgG throughout the study. In group G2, seroconversion began at 14 dpi (7.69%), increasing to 30.76% at 22 dpi, 53.64% at 28 dpi and 90.90% at 35 dpi, demonstrating a significant increasing trend in IgG detection. In contrast, G3 exhibited a less uniform dynamic, with 23% seroconversion at 22 dpi, 36.36% at 28 dpi and 45.45% at 35 dpi, suggesting a slower and less consistent progression. These results are corroborated by the model's fixed effects, where at 22, 28 and 35 dpi there was a significant increase in the S/P ratio, but the interactions of G2 and G3 with time were insignificant (Figure 2B).

**Figure 3:**
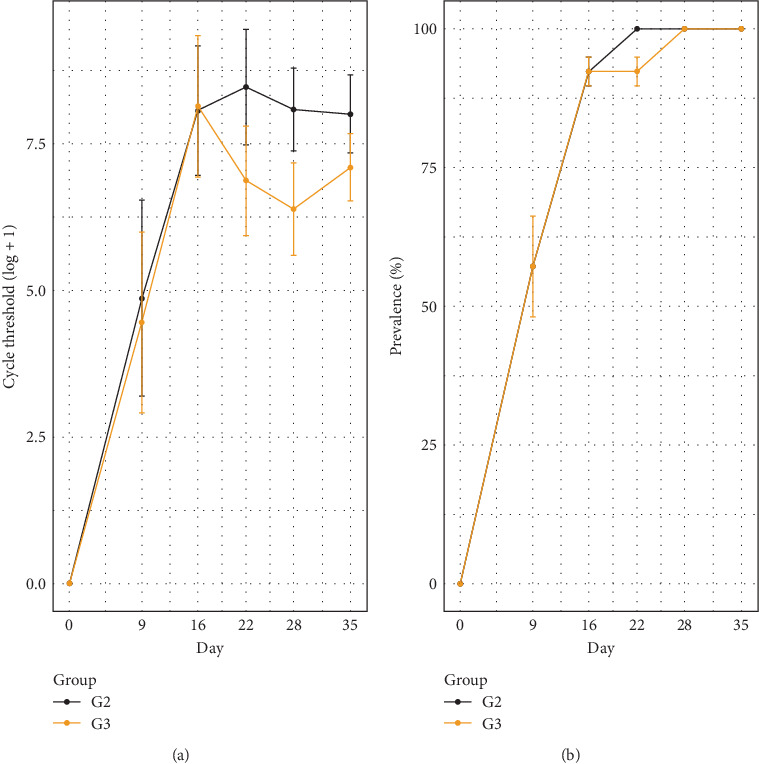
(A) Dynamics of *Mycoplasma hyopneumoniae* detection based on the amplification of a P102 gene fragment in laryngeal swabs from pigs in groups G2 and G3 following experimental inoculation. Detection was performed using quantitative real-time PCR (qPCR). (B) The lines represent the percentage (%) of qPCR-positive animals at each time point.

The analysis using a binomial linear model revealed a significant effect of experimental day on the qPCR results. However, the interactions between day and treatment group (G2 and G3) were not statistically significant, except at 28 dpi (*p*=0.043). This suggests that, while there are variations in qPCR over time and a trend towards reduction in group G3, these variations did not differ significantly between G2 and G3, except at 28 dpi (Figure 3A; Table 1).

Figure 3B presents the dynamics of *M. hyopneumoniae* detection in laryngeal swabs. G2 had detection rates of 57% (8/14), 92% (12/13), 100% (13/13), 100% (11/11) and 100% (11/11) at 9, 16, 22, 28 and 35 dpi, respectively. G3 experienced rates of 57% (8/14), 92% (12/13), 92% (12/13), 100% (11/11) and 100% (11/11) on the same days. Overall, G2 exhibited a slightly higher detection trend over time than G3, particularly at 22 dpi when detection in G3 was still at 92%. The only significant difference between groups occurred at 28 dpi, indicating that the interactions between days and groups were not consistently distinct throughout the analysed period (Figure 3; Table 1).

**Figure 4:**
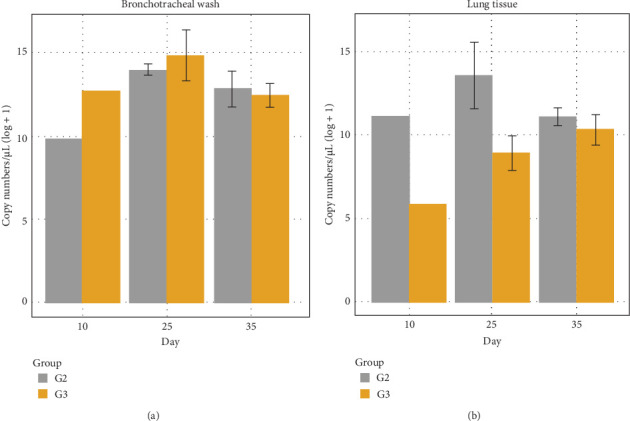
Dynamics of *Mycoplasma hyopneumoniae* detection based on quantification of P102 gene fragment copies in bronchoalveolar lavage fluid (BALF) (A) and lung tissue (B). Quantitative real-time PCR (qPCR) was used to assess bacterial load over time in the experimental groups.

No significant difference was observed between G2 and G3 in the detection of *M. hyopneumoniae* in BALF (Figure 4A). However, a significant difference between the two groups was observed in lung tissue at 25 dpi, with a significant reduction in G3 (Figure 4B; Table 1). All BALF and lung tissue samples tested positive by qPCR.

**Figure 5:**
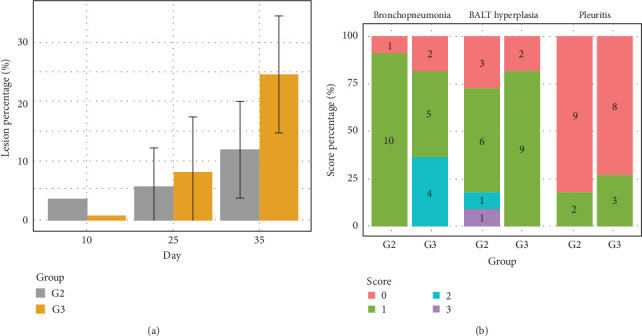
(A) Affected lung severity on a scale of 0% (no lesions) to 100% (entire lung affected). (B) Quantified microscopic lesions (bronchopneumonia, bronchus-associated lymphoid tissue hyperplasia and pleuritis). The distinct colours represent lesion scores 0 (normal), 1 (mild), 2 (moderate) and 3 (intense) [33, 43, 44].

The model suggests a marginally significant difference in total lesion scores between G2 and G3 at 35 dpi (*p*=0.0652), but the evidence is not strong (Supporting Information 3: Figure S1 and Figure 5A). Therefore, while there is an indication of a possible difference, further studies with a larger sample size or additional variables are needed to confirm this finding.

In the microscopic lesions, BALT hyperplasia was present in 75% (8/11) of G2 animals, bronchopneumonia in 90% (10/11) and pleuritis in 18.8% (2/11). BALT hyperplasia was detected in 81.81% (9/11) of G3 animals, bronchopneumonia in 81.81% (9/11) and pleuritis in 27% (3/11) (Figure 5B). Based on the logistic regressions, the treatment in G3 had a statistically significant effect on the presence of BALT hyperplasia, bronchopneumonia or pleuritis compared to G2. This indicates that, for these variables, the G3 treatment does not appear to influence the occurrence or severity of the assessed microscopic lesions compared to G2 (Figure 5B).

### 3.4. Evaluation of the Clinical Signs

**Figure 6:**
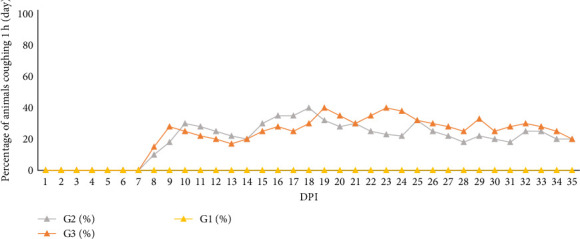
Graph showing the daily cough frequency (%) for each experimental group over the 35 days, with measurements taken for 1 h/day. The orange line represents G3, the grey line represents G2 and the yellow line represents G1. DPI, days post-inoculation.

Clinical signs of a non-productive, dry cough, characteristic of *M. hyopneumoniae* infection, were observed in all inoculated animals beginning at 7 dpi and persisting until the end of the study. Control animals did not exhibit fever or clinical signs resembling *M. hyopneumoniae* infection. Throughout the study, G2 piglets had 443 coughing episodes and G3 piglets had 464 coughing episodes over the 35-day study period.  Figure 6 displays the daily percentage of animals exhibiting a cough during the observation period, relative to the total number of animals in each group.

### 3.5. Differential Diagnosis

All animals tested negative for influenza A and *M. hyorhinis* by qPCR In G1, 50% (1/2) of the animals tested positive for *A. pleuropneumoniae*, *S. suis* and *P. multocida*. Piglets in G2 and G3 were 35.7% (5/14) positive for *G. parasuis* and *P. multocida* by qPCR. *A. pleuropneumoniae* was detected in 35.7% (5/14) of G2 animals and in 57.1% (8/14) of G3 animals.

### 3.6. Respiratory Microbiota Species Richness and Venn Diagram

**Figure 7:**
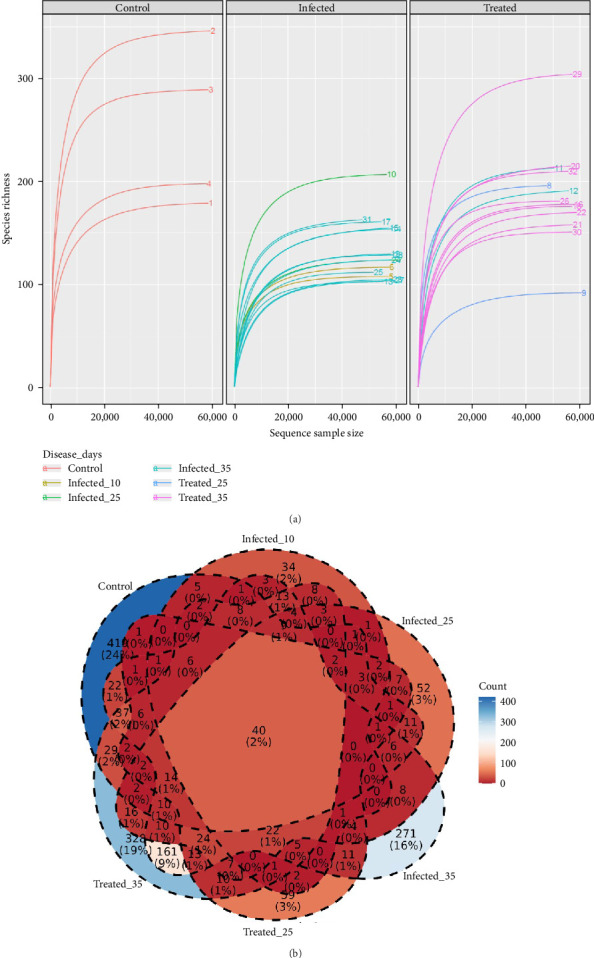
Bacterial species richness and venn diagrams of different taxonomic groups of the respiratory microbiota of pigs in different experimental groups: control (G1) healthy animals, infected (G2, at dpi 10, 25 and 35) with *M. hyopneumoniae* and treated (G3, dpi 25 and 35) with the antibiotic tylvalosin. (A) Rarefaction curves illustrating the species richness of ASVs (amplicon sequence variants) in the respiratory microbiota of pigs under different experimental conditions. The data are presented in three panels corresponding to each experimental group: control (negative control, G1, *n* = 4), infected (positive control, G2, *n* = 14) and treated (infected and treated, G3, *n* = 14). Each curve represents an individual animal within its respective group. The *x*-axis denotes the sequencing depth (number of sequences sampled), while the *y*-axis indicates the species richness (number of observed ASVs). Rarefaction analysis enables the evaluation of microbial diversity and sampling effort. A plateau in the curve suggests that the sequencing depth was sufficient to capture the microbial diversity present in that sample. (B) Venn diagram illustrating the shared ASVs among the experimental groups. Each circle represents a group, and the overlapping areas indicate shared ASVs. Percentages reflect the proportion of unique or shared ASVs relative to the total.

Analysis of microbial species richness (Figure 7) under the different treatment conditions and disease progression stages revealed the impact of infection and treatment on the indigenous microbiota of the animals' lungs, specifically in the lower respiratory tract. G1 exhibited consistently increasing species richness, indicating a larger microbial community than the other groups, despite having fewer samples (Figure 7A).

In contrast, G2 and G3 at various time points displayed reduced species richness, suggesting the potential loss of several bacterial species associated with infection and treatment (Figure 7A). Notably, G3 showed greater species richness compared to G2, especially at the last time point (35 dpi), suggesting that treatment may have contributed to the partial recovery of bacterial richness, but not necessarily with the return of the same species present before infection or treatment (Figure 7A). Furthermore, greater dispersion in species richness was observed among animals in G3, with some exhibiting higher and others lower numbers of species, without a discernible pattern. This phenomenon indicates the presence of microbiota dysbiosis, even after treatment.

Analysis of shared ASV using a Venn diagram revealed a clear distinction in bacterial species composition among the three experimental groups. Piglets in G1 had the highest number of unique ASVs, indicating a distinct microbial community from piglets in G2 and G3. G2 at 25 and 35 dpi shared the largest number of ASVs, suggesting a convergence in microbiota composition after a prolonged period of infection (Figure 7B).

Antibiotic treatment resulted in altered microbial composition, with G3 at 35 dpi sharing a significant number of ASVs with G1, indicating a trend towards partial recovery of the original bacterial microbiota. The intersection of all groups (Figure 7B), containing only 2% of the total ASVs, highlights the high interindividual variability in pig respiratory microbiota composition, as well as the influence of infection and treatment on bacterial ecology.

Another critical point is that both infection and treatment reduced the number of unique ASVs, demonstrating their significant impact on the indigenous microbiota in both situations. It is worth noting that the microbiota of healthy animals can contain more than 400 unique bacterial species. The evident increase in ASVs several days after infection and treatment demonstrates the resilience of this microbiota under adverse conditions and its capacity for recovery.

### 3.7. Microbiota Taxa Distribution Represented by ASV Density Plots

**Figure 8:**
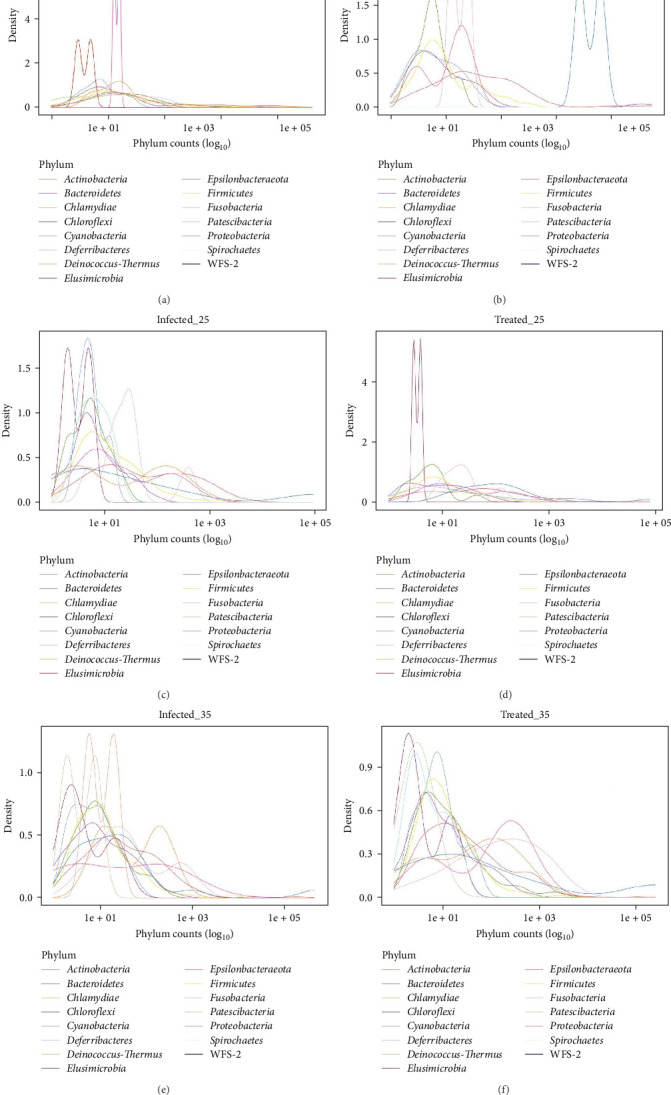
Distribution of amplicon sequence variants (ASVs) by bacterial phyla in the respiratory microbiota of pigs from different experimental groups: healthy control animals (G1), animals infected with *Mycoplasma hyopneumoniae* (G2) and infected animals treated with the antibiotic tylvalosin (G3). Each panel (A–F) presents a density plot showing the relative abundance (log_10_-transformed phylum counts) of bacterial phyla identified in bronchoalveolar lavage fluid (BALF) samples. The *x*-axis represents the abundance of each phylum, and the *y*-axis represents density. Panels are organised by experimental condition and time point: (A) G1-control; (B) G2-infected, 10 days post-infection (dpi); (C) G2-infected, 25 dpi; (D) G3-treated, 25 dpi; (E) G2-infected, 35 dpi; and (F) G3-treated, 35 dpi. Colours correspond to distinct bacterial phyla.

The plots in Figure 8 show the variation in the distribution of microbial phyla counts across the different experimental groups over time. On the *x*-axis, phyla counts are log_10_-transformed to accommodate the wide variation observed, while the *y*-axis represents the density of these counts, reflecting the abundance of each phylum. Analysis of microbiota composition revealed a significant shift in the relative abundance of bacterial phyla in response to experimental infection and treatment. G1 (Figure 8A) shows a lower number of ASVs compared to the other groups. Moreover, the phylum *Firmicutes* is clearly predominant, standing out as the most abundant bacterial group in G1, as evidenced by its pronounced peak compared to the other phyla. Additionally, the presence of two nearby peaks suggests the existence of two subgroups of *Firmicutes* with slightly different abundances. The phylum *Proteobacteria*, despite presenting a smaller density peak, is the second most abundant phylum. The remaining phyla, such as *Actinobacteria*, *Bacteroidetes*, *Chlamydiae*, *Chloroflexi*, *Cyanobacteria*, *Deferribacteres*, *Deinococcus-Thermus*, *Elusimicrobia*, *Epsilonbacteraeota*, *Fusobacteria*, *Patescibacteria*, *Spirochaetes* and WPS-2, show very low densities and more dispersed distributions, indicating they are less abundant in the respiratory microbiota of G1.

Infection induced a drastic increase in the abundance of *Tenericutes*, making it the dominant phylum in all infected groups, with abundance peaks for G2 at 10 and 25 dpi (Figure 8B,C). Simultaneously, there was a reduction in the counts of *Proteobacteria* in G2 at 10, 25 and 35 dpi (Figure 8B,C,E) and of *Bacteroidetes* in G2 at 10 and 25 dpi (Figure 8B,C). Antibiotic treatment modulated the microbiota composition, with different recovery patterns observed. In G3 at 25 dpi (Figure 8D), *Proteobacteria* showed a higher abundance peak than *Firmicutes*, while *Bacteroidetes* remained at low abundance. In contrast, G3 at 35 dpi (Figure 8F) exhibited *Firmicutes* as the most abundant phylum, followed by *Proteobacteria* and an increase in the abundance of *Bacteroidetes* compared to G2. These results demonstrate the impact of infection and treatment on the dynamics of the pig respiratory microbiota, with some phyla becoming more dominant and others decreasing in abundance, highlighting the complexity of the ecological dynamics of microbial communities in response to infection and antimicrobial therapies.

### 3.8. Assessment of Alpha and Beta Diversity of Pig Respiratory Microbiota

**Figure 9:**
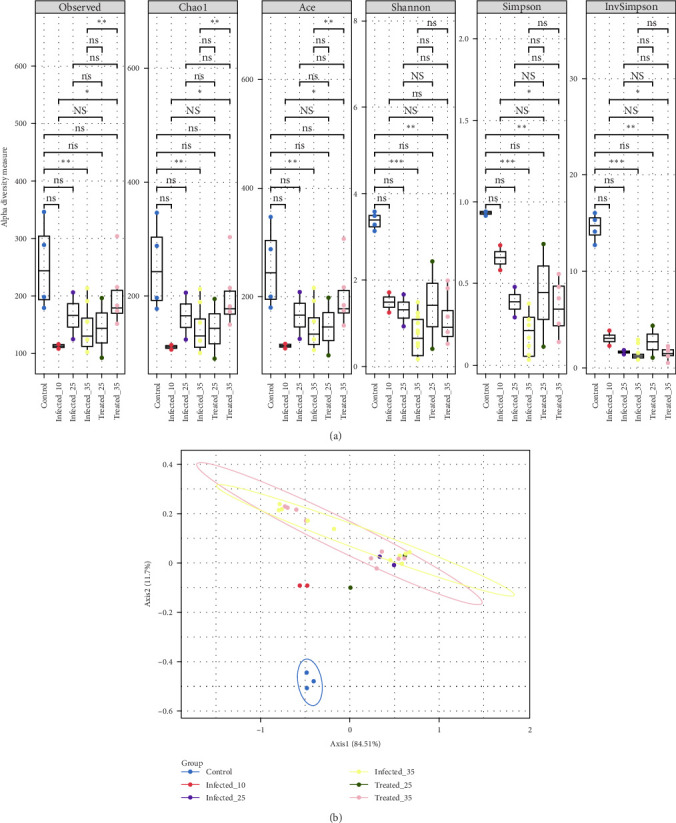
Alpha (A) and beta (B) diversity of the bacterial microbiota of the pig respiratory tract from different experimental groups: control (G1), infected (G2, at dpi 10, 25 and 35) and treated (G3, at dpi 25 and 35). (A) Alpha diversity of the pig respiratory microbiota in different experimental groups. Boxplots represent the variation in 6alpha indices: observed (observed richness), Chao1 (estimated richness), ACE (estimated richness), Shannon (diversity), InvSimpson (diversity) and Fisher (richness). Different letters above the boxplots indicate significant differences between groups (*p* < 0.05). (B) Beta diversity based on principal coordinates analysis (PCoA) of the pig respiratory microbiota demonstrates the dissimilarity between the microbial communities of the different experimental groups. Points represent individual samples, and the distance between points reflects the difference in microbiota composition. Ellipses represent the variation within each group.

The analysis of alpha diversity of the pig respiratory microbiota under various experimental scenarios and the statistical significance of the observed differences between groups is shown in Figure 9A. Species richness indices (observed, Chao1 and ACE) in G1 showed the highest microbial diversity across all evaluated metrics, indicating a healthy and balanced microbial community. In contrast, G2 at 10, 25 and 35 dpi exhibited a reduction in alpha diversity, which became more pronounced with longer infection time. However, this reduction was not statistically significant across all metrics when compared to G1 (*p* < 0.05). Even so, this suggests a negative impact of infection on microbial diversity.

Nevertheless, G3 at 25 and 35 dpi exhibited an increase in alpha diversity compared to G2, particularly 35 dpi, approaching the levels observed in G1. This suggests a partial recovery of the microbial community following treatment. Additionally, the analysis of diversity indices (Shannon and InvSimpson) revealed a similar pattern, showing significant differences between G2 and G1 (*p* < 0.05), while G3 did not differ significantly from G1. It is important to highlight that the absence of statistical significance between G1 and G3 does not preclude the possibility of biologically relevant differences.

The dissimilarity between microbial communities using the first two principal axes, which explain 86.28% of the total variance, is shown in Figure 9B. A clear separation is observed between G1 and the other two groups, evidenced by the distinct location of the points on the plot. G1 samples form a well-defined cluster in the lower-left corner, indicating high similarity among themselves and a different microbial composition compared to the other groups. G2 at 10, 25 and 35 dpi overlap in the upper-middle region of the plot, suggesting that infection alters the microbiota composition compared to G1, but the changes maintain similar profiles between the different infection time points. G3 at 25 and 35 dpi show greater dispersion on the plot, which suggests that the subdose treatment affects the microbiota composition slightly, but with variability in the individual responses of the animals and without a re-establishment of the microbiota to the healthy state of the controls.

### 3.9. Distribution of Absolute and Relative Abundances

**Figure 10:**
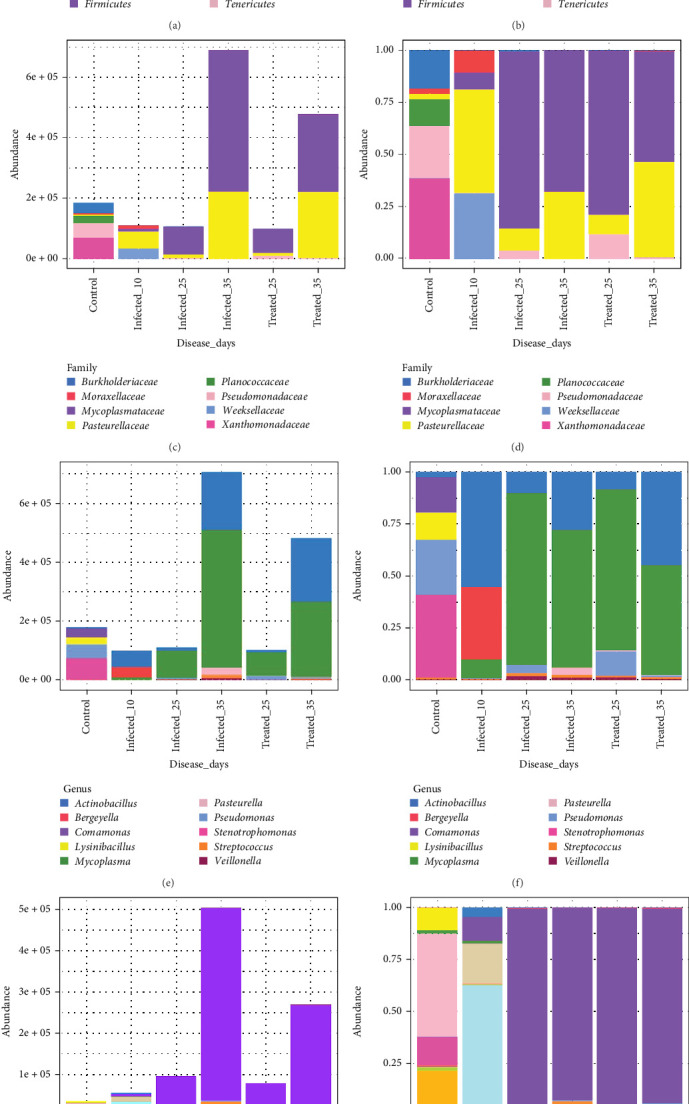
Absolute abundance (left) and relative abundance (right) of different taxonomic groups of the pig respiratory microbiota in six experimental groups: G1 control; G2 infected _10; G2 infected_ 25; G3 treatment_25; G3 infected_35 and G3 treatment_35. Each colour in the bars represents a different bacterium at its respective taxonomic level. The graphs show the microbiota composition at the taxonomic levels of phylum (A and B), family (C and D), genus (E and F) and species (G and H). Bars represent the abundance of each taxon in each experimental group.

Analysis of the pig respiratory microbiota at different taxonomic levels revealed significant alterations caused by experimental infection and subsequent treatment (Figure 10A–H). G1 exhibited a more diverse bacterial community, with a predominance of *Proteobacteria* (75%) and *Firmicutes* (20%) and the families *Xanthomonadaceae*, *Pseudomonadaceae*, *Pasteurellaceae* and *Burkholderiaceae*. The genera *Stenotrophomonas* and *Pasteurella* were predominant, followed by the species *Stenotrophomonas maltophilia*, *Sphingobacterium multivorum*, *Comamonas testosteroni* and *Comamonas jeotgali*.

Infections in G2 at 25 and 35 dpi resulted in a drastic reduction of *Proteobacteria* and an increase in *Firmicutes* and *Tenericutes*, with the latter becoming the dominant group. An increase in *Weeksellaceae* and a decrease in *Xanthomonadaceae* were also observed. In terms of genera, there was an increase in *Comamonas* and a decrease in *Stenotrophomonas*. The infection led to the dominance of *M. hyopneumoniae*, with a consequent reduction in other species, such as *Bergeyella zoohelcum*. This dominance extended to all taxonomic levels (*Tenericutes*, *Mycoplasmataceae* and *Mycoplasma*), severely impacting the microbiota structure, with *M. hyopneumoniae* representing almost 90% of the community and eliminating the presence of other taxa in the ASVs.

Treatment with tylvalosin in G3 reduced several dysbiotic taxonomic levels and diminished, albeit limitedly, the dominance of *M. hyopneumoniae* at 25 dpi. In G3 at 35 dpi, there was an increase in the number of *M. hyopneumoniae* ASVs, in addition to a small increase in the genus *Actinobacillus* sp., suggesting possible recolonisation of the lower respiratory tract by *M. hyopneumoniae* and *Actinobacillus* sp. simultaneously.

Although the treatment partially modulated the microbiota, it did not fully restore the original composition due to the subdosage and the short experimental period, which prevented complete recolonisation. In G3, *Tenericutes* decreased, while *Proteobacteria* and *Firmicutes* increased modestly. The dominance of *Mycoplasmataceae* was reduced, with an increase in *Pasteurellaceae* (G3 at 25 dpi) and *Planococcaceae* (G3 at 35 dpi). Although there was a reduction in *M. hyopneumoniae* load, this species remained dominant, suggesting that subtherapeutic antibiotic dosing is insufficient for effective pathogen control and may hinder the recovery of the overall microbiota post-infection.

### 3.10. Respiratory Biomarkers

**Figure 11:**
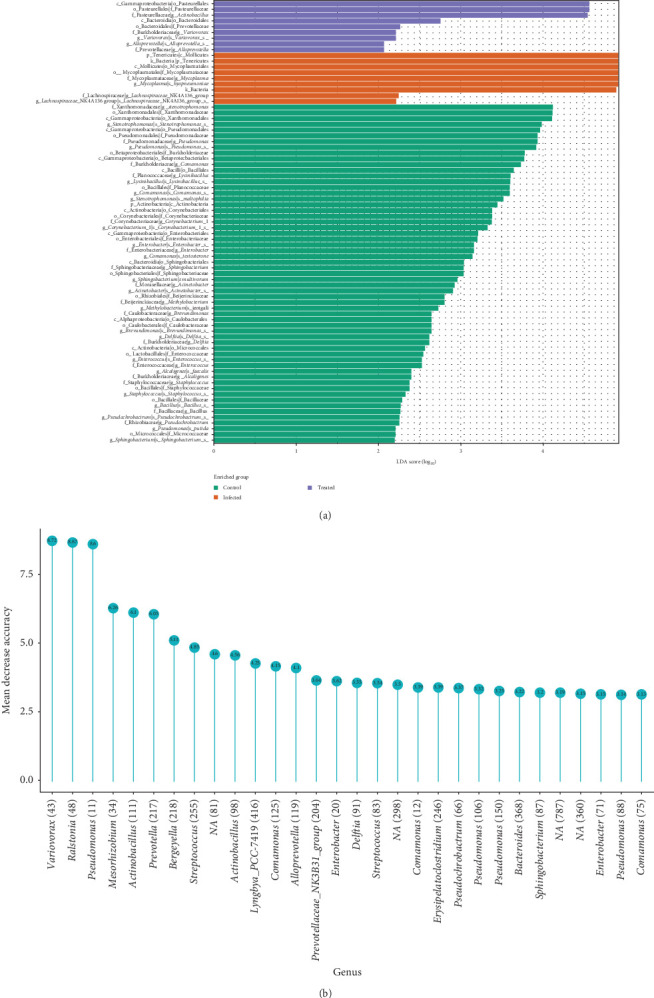
Respiratory biomarker analysis. (A) Differential enrichment analysis of the respiratory microbiota. The bar plot displays differentially abundant bacterial taxa between the control (green), infected (orange) and treated (purple) groups. The *x*-axis represents the LDA score (linear discriminant analysis) on a logarithmic scale (log10). The *y*-axis lists the taxa, organised by taxonomic level (p: phylum, c: class, o: order, f: family, g: genus, s: species). (B) Importance of bacterial genera in predicting respiratory health. The lollipop chart displays the importance of each bacterial genus in the ability of a predictive model to correctly classify the pig respiratory health status (control, infected and treated). The *y*-axis represents the mean decrease in model accuracy (mean decrease accuracy) when a given genus is removed. Genera with higher scores (longer lines) have greater predictive importance. In other words, these genera may serve as potential biomarkers, as their presence or absence strongly influences the model's ability to accurately classify the animal's respiratory health status. Numbers above the circles indicate the exact value of the mean decrease in accuracy. Numbers in parentheses after some genera may represent the number of ASVs associated with each genus. 'NA' indicates genera not classified.

The linear discriminant analysis effect size (LEfSe) analysis revealed significant differences in the taxonomic composition of the pig respiratory microbiota (Figure 11A) among the three experimental groups. G1 showed enrichment of several taxa belonging to *Proteobacteria* and *Bacteroidetes*, including *Xanthomonadaceae*, *Pseudomonadaceae* and *Weeksellaceae*, and the genera *Stenotrophomonas*, *Pseudomonas*, *Acinetobacter*, *Comamonas*, *Brevundimonas* and *Sphingobacterium*. At the species level, *Stenotrophomonas maltophilia*, *Sphingobacterium multivorum*, *Comonas testosteroni* and *Comonas jeotgali* were predominant in this group.

Infection with *M. hyopneumoniae* in G2 profoundly altered the microbiota composition, with a significant enrichment of *Tenericutes*, *Mycoplasmataceae*, *Mycoplasma* and *M. hyopneumoniae*, which became the dominant taxon. Concurrently, there was a reduction in the abundance of several dominant taxa in G1, including *S. maltophilia*.

Treatment with tylvalosin modulated the microbiota composition in G3 but did not fully restore it to the state observed in G1. While the relative abundance of *M. hyopneumoniae* was reduced in G3 compared to G2, this pathogen remained among the most abundant taxa. The treatment also led to the enrichment of certain taxa in Firmicutes, including the families *Planococcaceae* and *Streptococcaceae*, as well as the genera *Lysinibacillus* and *Streptococcus*. Additionally, *Lactobacillus delbrueckii* was enriched in this group.

The mean decrease accuracy metric in a random forest model (Figure 11B) identified the bacterial genera most predictive of pig respiratory health status. *Variivorax* had the highest influence on model accuracy (8.71 mean decrease in accuracy), followed by *Ralstonia* (8.37), *Pseudomonas* (8.11), *Mesorhizobium* (7.34), *Actinobacillus* (7.11), *Prevotella* (6.28), *Bergeyella* (6.17) and *Streptococcus* (5.25). The exclusion of these genera would result in the most significant drop in the model's predictive ability, suggesting their crucial role in distinguishing between the G1, G2 and G3.

Genera such as *Actinobacillus* (4.98), *Comamonas* (4.16 and 3.97), *Lyngbya* PCC-7419 (4.12) and *Alloprevotella* (4.19) also showed relevant, although smaller, contributions to model accuracy. *Erysipelatoclostridium*, *Pseudocrocabacterium*, *Bacteroides* and *Sphingobacterium* exhibited moderate importance (mean decrease in accuracy between 3.54 and 3.23). Other genera, including some not classified at the species level ('NA'), had less influence on prediction, with values below 3.

## 4. Discussion

This study investigated the efficacy of a subdose of tylvalosin, a third-generation macrolide, in treating EP in pigs, primarily caused by *M. hyopneumoniae*, and its impact on the respiratory microbiome. Tylvalosin is an antibiotic primarily used in veterinary medicine to treat bacterial infections in pigs and poultry [16, 17]. Tylvalosin was selected for its broad-spectrum activity against mycoplasmas and Gram-positive bacteria, with potential inhibition of some Gram-negative bacteria [13]. Due to growing concerns about antimicrobial resistance and the need for new therapeutic alternatives, tylvalosin has gained prominence as a safe and effective option.

Tylvalosin is especially effective against *M. hyopneumoniae*, exhibiting a significantly lower minimum inhibitory concentration compared to tylosin, another macrolide antibiotic. This makes tylvalosin an option for treating and preventing respiratory and enteric diseases in poultry and pigs, including mycoplasmosis, porcine ileitis, swine dysentery and necrotic enteritis, among other conditions [14, 16, 21, 22]. However, the use of subtherapeutic doses, whether due to unintentional errors in administration calculations or poor quality in the production of this medication, can be a problem in the selection of resistant microorganisms and the creation of dysbiosis in the microbiota of the animals undergoing treatment [25–27].

The negative control group (G1) in our study showed no clinical signs consistent with EP. There was a numerically greater variation in clinical signs in G3 compared to G2, but with no statistically significant difference. The onset of clinical signs is consistent with other findings [32] that characterised the UFV01 strain. The onset of coughing in experimentally infected pigs can be variable [82], occurring between 1- and 3-weeks post-infection.

G2 had 10% of lungs with lesions, while G3 had 25%. G1 had no lesions (0%) at 35 dpi (Figure 5 and S1). There is a trend towards a significant difference between G2 and G3. The pattern of lesions in group G2 was similar to that found [33] when characterising the UFV01 strain. In a study by Almeida et al. [41] using strain 232, considered highly virulent [83, 84], the mean lung lesion area at 28 dpi was 15.84%, a pattern also found by Toledo et al. [33]. However, in this study, G3 showed a significant increase in the degree of lesions, reaching 25%, potentially evidencing a worsening of the lesions when using a subdose of tylvalosin. The formation of macroscopic lesions and the clinical manifestations of the disease are multifactorial, involving immunopathological and environmental mechanisms [82, 85–87]. According to Ferraz et al. [2], each 1% increase in lesion area corresponds to a 1.8 g/day reduction in daily weight gain. Based on this reference, the negative impact on animal performance and respiratory health becomes evident.

Regarding the microscopic lesion scores (Figure 5B), G1 showed no lesions of any type. G2 and G3 exhibited pleuritis, bronchopneumonia and BALT hyperplasia, albeit with different frequencies. Lymphocyte recruitment and their mitotic capacity in lung tissue result in the lesion known as BALT hyperplasia [88], which is considered a typical lesion caused by *M. hyopneumoniae*. On the other hand, bronchopneumonia and pleuritis lesions result from secondary bacterial involvement, including agents such as *P. multocida*, *A. pleuropneumoniae*, *S. suis* and *G. parasuis* [3, 89, 90]. Although the primary focus of this study was to evaluate the impact of subclinical dosing of tylvalosin during *M. hyopneumoniae* infection, we acknowledge that the antibiotic may also influence the dynamics of other pathogens present in the respiratory tract. Rather than limiting the study, this aspect enhances its practical relevance, as it reflects conditions closer to those encountered in the field, where constant interactions occur between *M. hyopneumoniae* and other respiratory microorganisms.

Seroconversion, assessed by ELISA (Figure 2), was not uniform across the experimental groups. In G2 (infected only), seroconversion began at 14 days post-infection (dpi), reaching 90.9% at 35 dpi, consistent with previous studies using the same *M. hyopneumoniae* strain [33, 40]. In contrast, G3 (infected and treated with tylvalosin) exhibited a lower seroconversion rate of only 45.4% at 35 dpi.

Previous studies have shown that specific serum IgG antibodies against *M. hyopneumoniae* are typically detected 3–4 weeks after infection, peaking around 11–12 weeks and gradually declining thereafter [91]. Following infection, *M. hyopneumoniae* colonises the respiratory tract, triggering the humoral immune response and subsequent IgG production [92]. The markedly lower antibody response observed in G3 suggests that tylvalosin may modulate the humoral immune response, potentially through an immunomodulatory mechanism that reduces IgG production, even at subtherapeutic doses [18].

Tylvalosin is known to exert anti-inflammatory effects, including the suppression of pro-inflammatory cytokines, such as IL-8, IL-6, IL-1β, and TNF-α, as demonstrated in vitro and in vivo [18, 20, 21]. Although cytokine expression was not evaluated in our study, the reduction in antibody production in G3 may reflect an attenuated antigen presentation and a delayed activation of the humoral immune response due to tylvalosin's immunomodulatory activity.

While this anti-inflammatory effect may be beneficial in controlling EP—given that exacerbated inflammation contributes significantly to pulmonary lesions and clinical signs—it may also alter the timing and intensity of the host immune response. Thus, tylvalosin's role appears to extend beyond antimicrobial action, supporting the hypothesis of an immunomodulatory effect that warrants further investigation. Future studies comparing therapeutic and subtherapeutic dosing regimens will be essential to clarify the broader impacts of tylvalosin on immune dynamics.

Another hypothesis is that the reduced bacterial load observed in G3 may have resulted in lower antigenic stimulation and, consequently, a milder humoral response. Although bacterial quantification from laryngeal swabs increased over time in both groups, a significant difference between G2 and G3 was observed only at 28 dpi, with G3 showing a consistent trend toward lower bacterial loads. In qPCR analyses of BALF, no statistical difference was found between G2 and G3 (Figure 4A); however, in lung tissue, a significant reduction in bacterial load was detected in G3 at 25 dpi (Figure 4B). Importantly, all animals tested positive for *M. hyopneumoniae* in both sample types.

BALF samples were collected and sequenced in the V3/V4 region of the 16S rRNA gene. Quality-filtered reads were demultiplexed, and a total of 1,810,725 sequences were used for downstream analyses (mean = 56,584.375 ± standard deviation [SD] = 3252.11 reads/sample). Our study, using 16S rRNA gene amplicon sequencing, corroborates similar findings in the literature [10, 93, 94] by demonstrating the negative impact of *M. hyopneumoniae* infection on the pig respiratory microbiota. Recent evidence refutes the traditional view that healthy lungs are sterile, revealing the presence of a diverse lung microbiome with important implications for respiratory health [95].

The study of the pig respiratory microbiome is an emerging area with a significant impact on animal health [96]. This research field focuses on understanding the community of microorganisms present in the pig respiratory tract and how these communities influence animal health and welfare. *M. hyopneumoniae*, albeit not part of the core respiratory microbiota, modulates the native microbial community and promotes conditions that facilitate the colonisation and proliferation of other opportunistic pathogens [10], including those from transient and pathobiont populations.

Metagenomic and metataxonomic studies conducted previously have provided unprecedented insights into the taxonomic diversity, metabolic potential and ecological roles of microbial communities in biomes as diverse as the mammalian gastrointestinal and respiratory tracts [97, 98]. It is essential to elucidate the structure of the microbial community in the lungs and understand its role in maintaining lung health and preventing respiratory diseases [94].

As demonstrated by Almeida et al. [93] and Sonalio et al. [10], *M. hyopneumoniae* infection results in profound dysbiosis in the animal's respiratory microbiota, evidenced by a significant reduction in species richness (rarefaction curves and alpha diversity indices-Chao1, ACE and observed species) and the absolute dominance of the pathogen (Figures 9 and 10). Taxonomic analysis, in terms of relative and absolute abundance, demonstrated the ability of *M. hyopneumoniae* to multiply rapidly and dominate the lung microbiome and establish its niche, leading to the near disappearance of other species, as observed by Siqueira et al. [94] in lungs with lesions, where *M. hyopneumoniae* represented 95% of the reads. This dysbiosis pattern, consistent across all infected groups regardless of the time of infection, highlights the severe impact of the pathogen on the respiratory microbial ecosystem (Figure 10).

We identified bacterial genera and species, such as *Pasteurella*, in the respiratory microbiota that are jointly associated with the clinical manifestation of EP [4, 8, 15]. However, the presence of different pathogens in the respiratory tract—such as *P. multocida*, *S. suis*, *A. pleuropneumoniae* and *G. parasuis*—is not sufficient on its own to trigger the development of EP [5, 8–11]. These microorganisms generally act as secondary pathogens, whose colonisation and pathogenicity are facilitated by a primary infection by *M. hyopneumoniae*. Therefore, even when some of these agents are detected in clinically healthy animals, there is no evidence that, in the absence of *M. hyopneumoniae*, they are capable of independently initiating the clinical presentation characteristic of EP or PRDC. Tylvalosin treatment at the tested dose demonstrated a positive but incomplete effect on modulating the respiratory microbiota. Tylvalosin reduced the *M. hyopneumoniae* load, as observed through absolute abundance analysis (Figure 10), and allowed a slight increase in the abundance of other genera, corroborating the trend towards recovery of species richness observed in the rarefaction curves (Figure 7A), density plots and alpha diversity indices (Figure 9A). However, similar to the results of [93], microbiota recovery in BALF was not complete, with *M. hyopneumoniae* persisting as the dominant species.

The persistence of *M. hyopneumoniae* in high abundance even after treatment underscores the need to integrate relative and absolute abundance data to assess the true extent of microbiota recovery and the efficacy of interventions. Siqueira et al. [94] also observed the presence of *M. hyopneumoniae* in lungs without lesions, indicating the difficulty in eradicating the pathogen. Persistence may be attributed to resistance mechanisms, such as biofilm formation [83], cellular adhesion [82] and immunomodulation [99]. Persistent dysbiosis can have long-term impacts on pig respiratory health, predisposing them to secondary infections and compromising the resilience of the respiratory system.

Beta diversity (Figure 9B) revealed drastic alterations in the microbiota of infected, untreated animals (G2) compared to those of healthy animals (G1). Treated animals (G3) exhibited even more pronounced alterations than those in G2, but differed from G1, indicating the impact of infection [100] and the limited restoration of microbiota by the subdose antibiotic treatment. The aim of the antibiotic is to eliminate the pathogen and alleviate the clinical picture, which can affect other target microorganisms and indirectly impact symbionts or other organisms affected by the imbalance caused by infection and treatment [27].

The limited separation between treated and diseased animals in beta diversity analyses is common, especially when antibiotics significantly alter the microbiota [101, 102]. Studies in pigs [102] and mice [103] have shown that antibiotics can reduce microbial diversity and alter the composition of the gut microbiome, affecting the populations of *Firmicutes*, *Bacteroidetes* and *Proteobacteria*. In pigs, even with increased diversity in some treatments, Bray-Curtis's dissimilarity remained limited [102]. Antibiotic cocktails in mice disturbed microbiota, reducing the distinction between groups [103]. Microbiota resilience [104] and recovery time [105] are important factors.

G2 and G3 showed greater dissimilarity, while G1 exhibited high internal similarity and distance from the others. The high dissimilarity between G2 and G3 (compared to G1) confirms the impact of infection and the limited restoration by the subdose treatment, which neither differentiated G2 from G3 nor eliminated *M. hyopneumoniae*.

*M. hyopneumoniae* infection led to significant dysbiosis in the pig respiratory microbiota, characterised by the dominance of *M. hyopneumoniae* and a reduction in taxonomic diversity (Figure 11A). Tylvalosin treatment partially modulated the microbiota but did not completely reverse the dysbiosis. LEfSe analysis identified bacterial taxa that differentiate the groups, providing insights into the impact of infection and treatment. Genera, such as *Variivorax*, *Ralstonia* and *Pseudomonas* were shown to be influential and may be potential biomarkers for pig respiratory health and response to tylvalosin treatment. Their presence/absence is strongly associated with clinical outcome and therapeutic efficacy (Figure 11B).

Despite the valuable insights this study provides into the effects of tylvalosin on *M. hyopneumoniae* infection and the swine respiratory microbiota, several limitations should be acknowledged. First, the relatively small sample size particularly in the negative control group—may have reduced the statistical power to detect subtle differences between experimental groups [106]. However, this limitation did not compromise the ecological analyses, which benefit from the high-resolution nature of microbiota sequencing data and maintain robustness in assessing microbial community structures [107, 108]. Second, although the experimental design did not include a group treated with the approved therapeutic dose of tylvalosin, this methodological decision was intentional and aimed at specifically evaluating the effects of underdosing based on concentrations previously reported in the literature. Despite these constraints, the present study provides novel and relevant evidence on the clinical and microbial consequences of subtherapeutic tylvalosin exposure, and highlights the potential of the respiratory microbiota as a biomarker for health status and treatment response. These findings reinforce the urgent need for more targeted and responsible antimicrobial stewardship strategies in swine production.

Future studies may build upon these findings by investigating the prevalence and underlying causes of subdosage in swine production systems, aiming to improve the accuracy and consistency of antimicrobial administration. Comparative trials between therapeutic and subtherapeutic regimens, as well as analyses of microbiota resilience over time, will be essential for developing responsible antibiotic use strategies in veterinary medicine. A deeper understanding of the factors leading to inconsistent dosing—such as delivery systems, variability in feed distribution, or operational challenges—could clarify the conditions that favour the persistence and potential selection of *M. hyopneumoniae* strains with reduced antimicrobial susceptibility. The experimental model employed in this study, which combines controlled in-feed tylvalosin exposure with respiratory microbiota profiling, offers a robust framework for evaluating therapeutic strategies. It also supports the development of targeted interventions to mitigate antimicrobial resistance and improve respiratory health and productivity in pig farming.

## 5. Conclusion

This study demonstrates that subtherapeutic administration of tylvalosin at 1.0625 mg/kg/day is insufficient to eliminate *M. hyopneumoniae* in pigs and fails to prevent the development of typical clinical signs, lung lesions and respiratory microbiota dysbiosis. Although partial modulation of the microbiota and a reduction in bacterial load were observed, the pathogen persisted and microbial diversity remained altered. The antibiotic appeared to attenuate the humoral immune response, potentially through its immunomodulatory effects, and contributed to a limited recovery of the respiratory ecosystem. These findings reinforce the need to avoid subtherapeutic antibiotic dosing in swine production, as it may promote pathogen persistence, dysbiosis and antimicrobial resistance. Moreover, this work highlights the importance of integrating microbiome analysis into the evaluation of therapeutic efficacy and respiratory health. Our evidence is crucial to strengthen regulations in countries where the practice of subtherapeutic antimicrobial use still persists. It also alerts producers to the risks of unsupervised dosage adjustments. Our findings strongly suggest that subtherapeutic antibiotic use in pigs should be avoided.

## Figures and Tables

**Table 1:** Mean and standard deviation values of *Mycoplasma hyopneumoniae* P102 gene copies (expressed as copies/µL) detected by quantitative PCR (qPCR) in laryngeal swabs, bronchoalveolar lavage fluid (BALF) and lung tissue samples collected from animals in the G2 (positive control) and G3 (infected and treated) groups.

Group	Swab							BALF	Tissue	BALF	Tissue	BALF	Tissue
		Day 0	9 dpi	16 dpi	22 dpi	28 dpi	35 dpi	10 dpi		25 dpi		35 dpi	
G2	Average	0	9.53E + 03	9.47E + 03	3.45E + 03	1.95E + 03	2.45E + 03	1.82E + 04	6.98E + 04	1.27E + 06	2.35E + 06	3.42E + 05	8.94E + 04
	DP	0	1.07E + 04	2.45E + 04	1.13E + 05	2.76E + 04	1.17E + 04	2.12E + 02	4.90E + 03	3.83E + 05	2.54E + 06	2.15E + 06	1.04E + 05
	Number	0/14	8/14	12/13	13/13	11/11	11/11	1/1	1/1	2/2	2/2	11/11	11/11
	%	0	57.14	92.30	100	100	100	100	100	100	100	100	100

G3	Average	0	1.82E + 03	6.18E + 03	1.18E + 03	5.00E + 02	1.73E + 03	3.33E + 05	3.55E + 02	5.43E + 06	8.60E + 03	2.23E + 05	3.00E + 04
	DP	0	9.86E + 03	1.26E + 05	8.72E + 03	1.15E + 04	3.42E + 03	7.07E + 03	9.48E + 00	5.68E + 06	1.07E + 04	9.80E + 05	4.43E + 05
	Number	0/14	8/14	12/13	12/13	11/11	11/11	1/1	1/1	2/2	2/2	11/11	11/11
	%	0	57.14	92.30	92.30	100	100	100	100	100	100	100	100

*Note:* Samples were collected at various time points post-infection (dpi). For each matrix and time point, the table shows: (i) mean gene copy number; (ii) standard deviation (SD); (iii) number of positive animals versus total sampled (number) and (iv) percentage of qPCR-positive animals (%). Swab samples were collected on days 0, 9, 16, 22, 28 and 35 dpi. BALF and lung tissue samples were obtained at 10, 25 and 35 dpi from subsets of euthanised animals.

## Data Availability

Any data not presented here will be made available upon reasonable request.

## Supporting Information

Supporting Information 1 Table S1. Clinical evaluation of animals. Rectal temperature, heart rate, and respiratory rate of animals according to different treatments and assessment days.

Supporting Information 2 Table S2. Complete blood count of animals included in the study.

Supporting Information 3 Figure S1. Photographs of the lungs from groups G2 infected_10 (1 and 2), G2 infected_25 (3 and 4), G2 treated_25 (5 and 6), control G1 (7 and 8), G3 infected_35 (9–19), and G3 treated_35 (20–30).
