# Supplementary material for: Use of Subtherapeutic Tylvalosin Against Mycoplasma hyopneumoniae: Implications For Respiratory Microbiome Dysbiosis and Swine Lung Health
Source: Transbound Emerg Dis. 2025 Aug 18;2025:8903237. doi: 10.1155/tbed/8903237 (PMC12377971; doi:10.1155/tbed/8903237)
Supplement: Supporting Information 1 — Table S1. Clinical evaluation of animals. Rectal temperature, heart rate, and respiratory rate of animals according to different treatments and assessment days. [file 8903237.f1.docx]

Table S1. Clinical evaluation does animals. Rectal temperature, heart rate, and respiratory rate of animals according to different treatments and assessment days.

| **Days** | **Rectal temperature (°C)** | | |
| --- | --- | --- | --- |
|  | **G1** | **G2** | **G3** |
| **D0** | 39.1 ± 0.38 ^aA^ | 39.0 ± 0.14 ^aA^ | 39.1 ± 0.14 ^aA^ |
| **D+9** | 39.4 ± 0.36 ^aA^ | 39.0 ± 0.14 ^aA^ | 39.3 ± 0.13 ^aA^ |
| **D+16** | 39.5 ± 0.36 ^aA^ | 39.3 ± 0.14 ^aA^ | 39.3 ± 0.13 ^aA^ |
| **D+22** | 39.7 ± 0.36 ^aA^ | 39.1 ± 0.14 ^aA^ | 39.0 ± 0.14 ^aB^ |
| **D+28** | 39.8 ± 0.38 ^aA^ | 39.5 ± 0.16 ^aA^ | 39.8 ± 0.16 ^aA^ |
| **D+35** | 39.4 ± 0.38 ^aA^ | 39.5 ± 0.16 ^aA^ | 39.8 ± 0.16 ^aA^ |
|  | **Frequency Cardiac (bpm)** | | |
| **D0** | 100 ± 11.75 ^aA^ | 109 ± 4.29 ^aA^ | 112 ± 4.29 ^aA^ |
| **D+9** | 82 ± 11.75 ^aA^ | 101 ± 4.44 ^aB^ | 109 ± 4.29 ^aA^ |
| **D+16** | 78 ± 11.75 ^bA^ | 119 ± 4.61 ^aA^ | 113 ± 4.44 ^aA^ |
| **D+22** | 88 ± 11.75 ^aA^ | 101 ± 4.61 ^aA^ | 105 ± 4.61 ^aA^ |
| **D+28** | 70 ± 11.75 ^aA^ | 109 ± 5.01 ^aA^ | 106 ± 5.01 ^aA^ |
| **D+35** | 104 ± 11.75 ^aA^ | 104 ± 5.01 ^aA^ | 119 ± 5.01 ^aA^ |
|  | **Frequency Respiratory (mpm)** | | |
| **D0** | 72.0 ± 11.56 ^aA^ | 81.3 ± 4.22 ^aA^ | 76.0 ± 4.22 ^aA^ |
| **D+9** | 61.0 ± 11.56 ^aA^ | 63.0 ± 4.37 ^aA^ | 62.9 ± 4.22 ^aA^ |
| **D+16** | 59.0 ± 11.56 ^aA^ | 72.1 ± 4.53 ^aA^ | 72.6 ± 4.37 ^aA^ |
| **D+22** | 68.0 ± 11.56 ^aA^ | 51.0 ± 4.53 ^aB^ | 70.2 ± 4.53 ^aA^ |
| **D+28** | 52.0 ± 11.56 ^aA^ | 62.7 ± 4.93 ^aA^ | 75.6 ± 4.92 ^aA^ |
| **D+35** | 56.0 ± 11.56 ^aA^ | 58.7 ± 4.93 ^aA^ | 79.6 ± 4.92 ^aA^ |

Different lowercase letters within a row (between groups at a given time point) and different uppercase letters within a column (between time points for a given group) indicate significant differences by Tukey's test (p < 0.05). Reference values are according to [36].
