# Supplementary material for: Use of Subtherapeutic Tylvalosin Against Mycoplasma hyopneumoniae: Implications For Respiratory Microbiome Dysbiosis and Swine Lung Health
Source: Transbound Emerg Dis. 2025 Aug 18;2025:8903237. doi: 10.1155/tbed/8903237 (PMC12377971; doi:10.1155/tbed/8903237)
Supplement: Supporting Information 2 — Table S2. Complete blood count of animals included in the study. [file 8903237.f2.docx]

Table S2. Complete blood count of animals included in the study

| **Erythrogram** | **G1** | **G2** | **G3** |
| --- | --- | --- | --- |
| **Erythrocytes (10^6^ /µl)** | 6,08 ± 0,37 ^a^ | 5,94 ± 0,13 ^a^ | 5,91 ± 0,13 ^a^ |
| **Hemoglobin (g/dl)** | 11,2 ± 0,61 ^a^ | 10,2 ± 0,22 ^a^ | 10,7 ± 0,22 ^a^ |
| **Hematocrit (%)** | 35,0 ± 1,83 ^a^ | 31,8 ± 0,67 ^a^ | 33,3 ± 0,67 ^a^ |
| **CHCM (%)** | 31,9 ± 0,41 ^a^ | 32,1 ± 0,15 ^a^ | 32,2 ± 0,15 ^a^ |
| **VCM (fl)** | 57,5 ± 2,87 ^a^ | 53,8 ± 1,05 ^a^ | 56,4 ± 1,05 ^a^ |
| **Eosinophils (cels/μl)** | 135,5 ± 23,5 ^a^ | 109,5 ± 28,4 ^a^ | 204,1 ± 60,6 ^a^ |
| **Leukocytes (cels/μl)** | 13550 ± 3114 ^a^ | 13700 ± 1137 ^a^ | 12980 ± 1137 ^a^ |
| **Basophils (cels/μl) *** | 0.0 ^a^ | 32,1 ± 22,4 ^a^ | 21,1 ± 15,4 ^a^ |
| **Rods (cels/μl)** | 56,0 ± 56,6 ^a^ | 43,7 ± 20,7 ^a^ | 35,1 ± 20,7 ^a^ |
| **Monocytes (cels/μl) *** | 0,0 ^a^ | 71,7 ± 33,4 ^a^ | 41,5 ± 24,7 ^a^ |
| **Segmented (cels/μl)** | 6204 ± 2116 ^a^ | 4815 ± 773 ^a^ | 4795 ± 773 ^a^ |
| **Lymphocytes (cels/μl)** | 7154 ± 1712 ^a^ | 8628 ± 625 ^a^ | 7883 ± 625 ^a^ |
| **Platelets (/μl)** | 189500 ± 64129 ^a^ | 272600 ± 23416 ^a^ | 254200 ± 23416 ^a^ |
| **Biochemical** | **G1** | **G2** | **G3** |
| **Alanine aminotransferase (U/L)** | 67,5 ± 5,27 ^a^ | 52,9 ± 1,92 ^b^ | 54,3 ± 1,92 ^b^ |
| **Aspartate aminotransferase (U/L)** | 69 ± 14,8 ^a^ | 66 ± 5,4 ^a^ | 73 ± 5,4 ^a^ |
| **Alkaline phosphatase (U/L)** | 652 ± 92,2 ^a^ | 637 ± 33,7 ^a^ | 651 ± 33,7 ^a^ |
| **Total bilirubin (mg/ dl)** | 0,26 ± 0,06 ^a^ | 0,19 ± 0,02 ^a^ | 0,17 ± 0,02 ^a^ |
| **Direct bilirubin (mg/ dl)** | 0,13 ± 0,03 ^a^ | 0,05 ± 0,01 ^b^ | 0,06 ± 0,01 ^b^ |
| **Indirect bilirubin (mg/ dl)** | 0,14 ± 0,06 ^a^ | 0,14 ± 0,02 ^a^ | 0,11 ± 0,02 ^a^ |
| **Glutamyl transferase (U/L)** | 52,0 ± 9,88 ^a^ | 49,1 ± 3,61 ^a^ | 45,4 ± 3,61 ^a^ |
| **Albumin (g/dl)** | 2,70 ± 0,27 ^a^ | 2,53 ± 0,10 ^a^ | 2,52 ± 0,10 ^a^ |
| **Globulin (g/dl)** | 1,35 ± 0,30 ^a^ | 1,95 ± 0,11 ^a^ | 1,93 ± 0,11 ^a^ |
| **Proteins (g/dl)** | 4,13 ± 0,35 ^a^ | 4,59 ± 0,13 ^a^ | 4,54 ± 0,13 ^a^ |
| **Creatinine (g/dl)** | 1,60 ± 0,19 ^a^ | 1,64 ± 0,07 ^a^ | 1,57 ± 0,07 ^a^ |
| **Urea (mg/dl)** | 12,0 ± 3,44 ^a^ | 11,6 ± 1,25 ^a^ | 14,7 ± 1,25 ^a^ |

Different lowercase letters within a row indicate significant differences between groups by Tukey's test (p < 0.05). VCM = mean corpuscular volume; CHCM = mean corpuscular haemoglobin concentration.
