# Supplementary figures and images for: Use of Subtherapeutic Tylvalosin Against Mycoplasma hyopneumoniae: Implications For Respiratory Microbiome Dysbiosis and Swine Lung Health
Source: Transbound Emerg Dis. 2025 Aug 18;2025:8903237. doi: 10.1155/tbed/8903237 (PMC12377971; doi:10.1155/tbed/8903237)

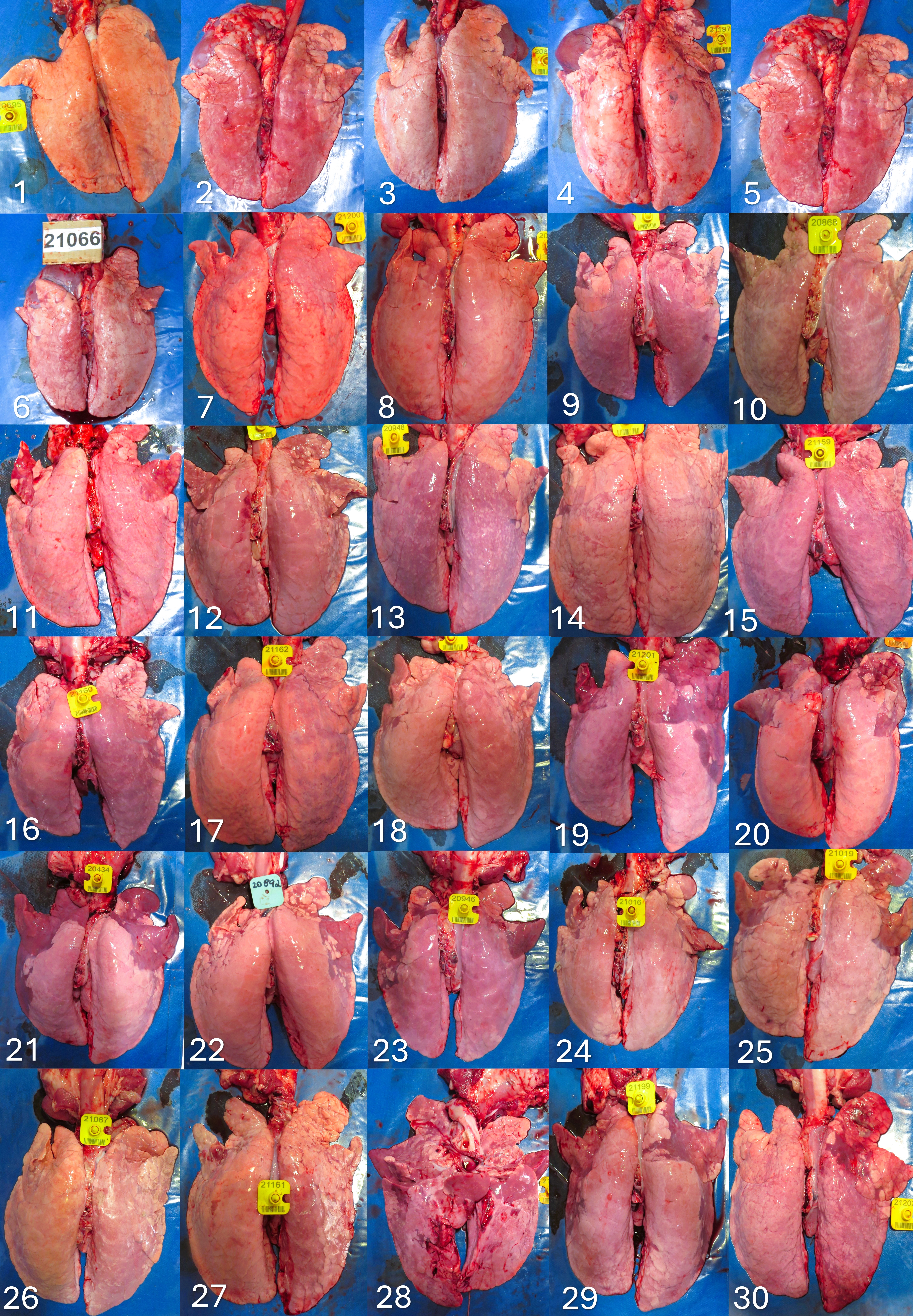

Supplement: Supporting Information 3 — Figure S1. Photographs of the lungs from groups G2 infected_10 (1 and 2), G2 infected_25 (3 and 4), G2 treated_25 (5 and 6), control G1 (7 and 8), G3 infected_35 (9–19), and G3 treated_35 (20–30). [file 8903237.f3.jpg]
